# One-pot production of butyl butyrate from glucose using a cognate “diamond-shaped” *E. coli* consortium

**DOI:** 10.1186/s40643-021-00372-8

**Published:** 2021-02-21

**Authors:** Jean Paul Sinumvayo, Chunhua Zhao, Guoxia Liu, Yin Li, Yanping Zhang

**Affiliations:** 1 CAS Key Laboratory of Microbial Physiological and Metabolic Engineering, State Key Laboratory of Microbial Resources, Institute of Microbiology, Chinese Academy of Sciences, Beijing, 100101 China; 2 University of Chinese Academy of Sciences, Beijing, 100049 China

**Keywords:** Butyl butyrate, *E. coli*, Cognate consortium, Butanol, Butyrate

## Abstract

Esters are widely used in plastics, textile fibers, and general petrochemicals. Usually, esters are produced via chemical synthesis or enzymatic processes from the corresponding alcohols and acids. However, the fermentative production of esters from alcohols and/or acids has recently also become feasible. Here we report a cognate microbial consortium capable of producing butyl butyrate. This microbial consortium consists of two engineered butyrate- and butanol-producing *E*. *coli* strains with nearly identical genetic background. The pathways for the synthesis of butyrate and butanol from butyryl-CoA in the respective *E. coli* strains, together with a lipase-catalyzed esterification reaction, created a “diamond-shaped” consortium. The concentration of butyrate and butanol in the fermentation vessel could be altered by adjusting the inoculation ratios of each *E. coli* strain in the consortium. After optimization, the consortium produced 7.2 g/L butyl butyrate with a yield of 0.12 g/g glucose without the exogenous addition of butanol or butyrate. To our best knowledge, this is the highest titer and yield of butyl butyrate produced by *E. coli* reported to date. This study thus provides a new way for the biotechnological production of esters. 
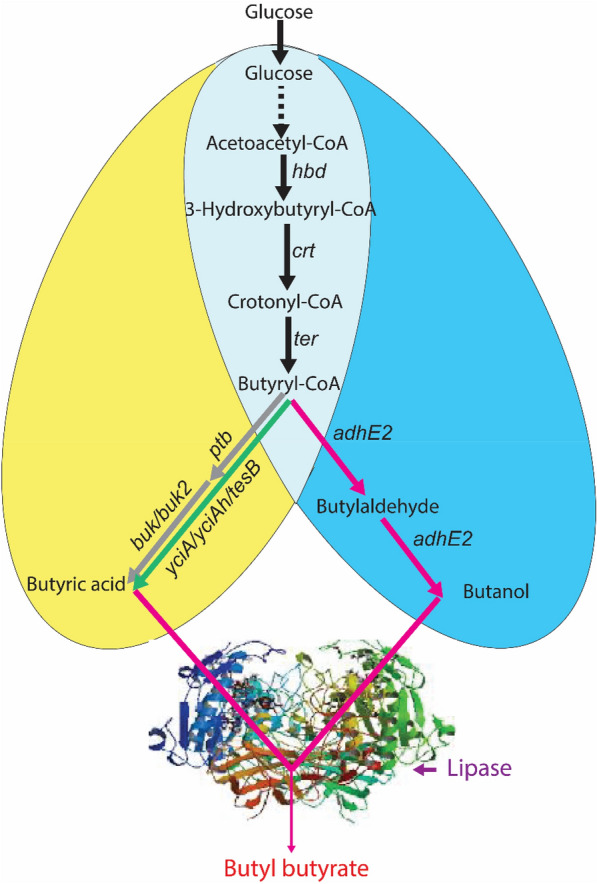

## Introduction

Fatty acid esters are a large group of value-added chemicals derived from short-chains alcohols and carboxylic acids. They are present in natural sources such as flowers, fermented beverages, and particularly in fruits (Chung et al. 2015; Jenkins et al. 2013). Notably, butyl butyrate (BB) is known as a flavor and fragrance compound that is widely used in foods, beverages, perfumes, and cosmetics (Santos et al. 2007). BB is also an important solvent widely used in the production of plastics, fibers, and processing of petroleum products (Horton and Bennett 2006; Matte et al. 2016).

Like most esters (R1COOR2), BB is traditionally produced by esterification of butyrate and butanol, which is usually conducted using inorganic catalysts at relatively high temperatures (Ju et al. 2011; Kang et al. 2011), but an enzymatic process for the production of BB has also been developed (Van den Berg et al. 2013; Matte et al. 2016). While current catalytic and enzymatic BB production processes all require the external supplementation of butanol and butyrate, some *Clostridium* species are able to produce butyrate, and can further convert the produced butyrate into butanol. However, most of the butyrate produced during acetone-butanol-ethanol fermentation is converted into butanol, leaving insufficient butyrate available for the esterification reaction. Therefore, butyrate, butanol, or both, need to be added to maintain sufficient levels of precursors (Xin et al. 2019). For example, 7.9 g/L butyrate had to be supplemented to a fed-batch fermentation of xylose by *Clostridium* sp. strain BOH3 to produce 22.4 g/L BB (Xin et al. 2016), while 10 g/L butanol needed to be added to a fermentation of *Clostridium tyrobutyricum* to achieve a BB titer of 34.7 g/L (Zhang et al. 2017).

Recently, Cui et al. developed a clostridial consortium comprising the butanol-producing *C. beijerinckii* and the butyrate-producing *C. tyrobutyricum*. They demonstrated that this consortium could produce 5.1 g/L BB without the addition of exogenous substrates. However, the yield of BB (0.068 g/g) was rather low, most likely due to the imbalanced ratio of butanol and butyrate, as well as the production of byproducts such as acetone and isopropanol (Cui et al. 2020). The imbalanced ratio of butanol and butyrate may be exacerbated by the two different species in the clostridial co-culture, with disparate optimal growth conditions. To solve this, we proposed a cognate microbial consortium for BB production, comprising butyrate- and butanol-producing *E. coli* strains with the same genetic background. If such a pair of cognate *E. coli* strains could be developed, their nearly identical genetic background would allow them to achieve a balanced production of butyrate and butanol by simply adjusting the composition of each strain in the consortium, thus more efficient BB production.

**Fig. 1:**
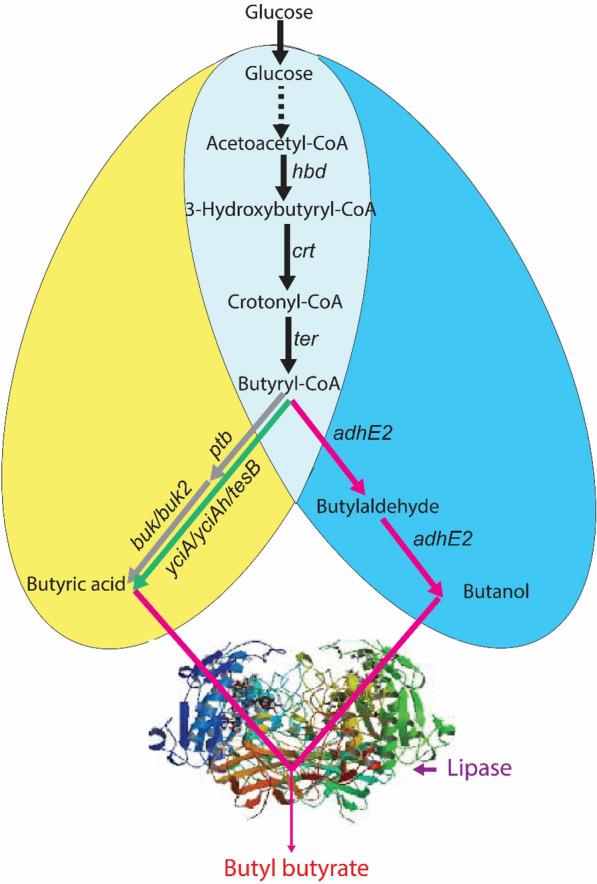
Construction of a “diamond-shaped” consortium for the direct production of butyl butyrate from glucose using two *E. coli* strains with the same genetic background. The butyric acid-producing strain is shown in yellow, and the butanol-producing strain in blue. A shared pathway from glucose to butyryl-CoA is shown in light grey in overlap between the two strains. A green arrow indicates the thioesterase pathway (encoded by *yciA, yciAh* or *tesB*), while the *ptb*-*buk* and *ptb*-*buk2* route is indicated by a grey arrow. The production routes of precursors (butanol and butyric acid) and their conversion into butyl butyrate with the assistance of lipase enzyme forms a “diamond-shaped” structure, which was used to define the consortium. The genes *hbd* (3-hydroxybutyryl-CoA dehydrogenase), *crt* (crotonase), *adhE2* (aldehyde/alcohol dehydrogenase), *ptb* (phosphate butyryltransferase), as well as *buk* and *buk2* (butyrate kinase) are derived from *C. acetobutylicum*; the *ter* gene (trans-enoyl-CoA reductase) is from *Treponema denticola; yciA* and *tesB* (acyl-CoA thioesterase) are from *E. coli*; *yciAh* (acyl-CoA thioesterase) is from *Haemophilus influenza*

Previously, we developed the chromosomally engineered *E. coli* strain EB243 capable of efficiently producing butanol from glucose (Dong et al. 2017). We intended to construct another butyrate-producing *E. coli* strain by redirecting the carbon flow at the node of butyl-CoA, thus shifting the carbon flow from butanol to butyrate production. When both strains were co-cultured and supplied with lipase, an *E. coli* consortium capable of directly producing BB from glucose can be constructed (Fig. 1). In this consortium, the two engineered *E. coli* strains share the same upstream metabolism, which diverges at the butyryl-CoA node and re-converges at BB, thus forming a “diamond-shaped” consortium (Fig. 1). We demonstrate the feasibility of using such a homogeneous microbial consortium for the production of esters with the assistance of exogenously added lipase in a two-liquid-phase fermentation system, providing a new approach for the biotechnological production of esters.

## Materials and methods

### Strains, plasmids and primers

*E. coli* EB243 (Dong et al. 2017) was used as the starting strain for metabolic engineering. All strains and plasmids used in this study are listed in Table 1. All primers (Additional file 1: Table S1) were synthesized by Invitrogen (Beijing, China) and purified via polyacrylamide gel electrophoresis. Candidate genes encoding acyl-CoA thioesterase (*yciA*, *tesB*) were amplified by PCR from the genomic DNA of *E. coli* BW25113, while those encoding phosphate butyryltransferase (*ptb*) and butyrate kinase (*buk* and *buk2*) were amplified from the genome of *Clostridium acetobutylicum* DSM 1731. The codon-optimized *yciAh* variant of the acyl-CoA thioesterase gene *yciA* from *Haemophilus influenzae* (Menon et al. 2015) was synthesized by GenScript (Nanjing, China). Subsequently, each gene was cloned into the pAC2 plasmid under the control of the miniPtac promoter (Zhao et al. 2019) and independently expressed in strain EB243ΔadhE2, resulting in the plasmids and strains summarized in Table 1.

**Table 1:** Strains and plasmids used in this study

Strain or plasmid	Relevant characteristics	Reference or source
Strains		
* E. coli* EB243	Derived from BW25113; Containing butanol synthesis pathway genes (*atoB*, *hbd*, *crt*, *ter*, *adhE2*) and *fdh*, as well as deletions of a*dhE*, *eutE*, *yqhD*, *ackA*, *pta*, *hyc-hyp*, *fdhF*, *poxB*, *pck*, *fumB*, *fumAC*, *tdcD*, *mdh*, *focA*, *ppc*, *mgsA*, *yieP*, *stpA*, *yqeG*, and *yagM*	(Dong et al. 2017)
* E. coli* EB243ΔadhE2	EB243 derivative, with *adhE2* deleted	This study
* E. coli* EB243ΔadhE2*-*pAC2	EB243 derivative, harboring the plasmid pAC2	This study
* E. coli* EB243ΔadhE2-pAC2-ptb-buk	EB243 derivative, harboring the plasmid pAC2-ptb-buk	This study
* E. coli* EB243ΔadhE2*-*pAC2-ptb-buk2	EB243 derivative, harboring the plasmid pAC2-ptb-buk2	This study
* E. coli* EB243ΔadhE2*-*pAC2-yciAh	EB243 derivative, harboring the plasmidpAC2-yciAh	This study
* E. coli* EB243ΔadhE2*-*pAC2-tesB	EB243 derivative, harboring the plasmid pAC2-tesB	This study
* E. coli* EB243ΔadhE2*-*pAC2-yciA	EB243 derivative, harboring the plasmid pAC2-yciA	This study
*E*. *coli* EB243ΔadhE2::yciAh	Derived from EB243ΔadhE2; with the synthetic thioesterase gene “yciAh” integrated into the chromosome	This study
Plasmids		
pAC2	pACYC184 derivative, miniPtac, *cat,* Kan^R^	(Zhao et al. 2019)
pAC2-ptb-buk	pAC2 derivative, expressing the *ptb*-*buk* genes	This study
pAC2-ptb-buk2	pAC2 derivative, expressing the *ptb*-*buk2* genes	This study
pAC2-yciAh	pAC2 derivative, expressing the *yciAh* gene	This study
pAC2-yciA	pAC2 derivative, expressing the *yciA* gene	This study
pAC2-tesB	pAC2 derivative, expressing the *tesB* gene	This study
pTargetF	*aadA*, guide RNA transcription	(Jiang et al. 2015)
pCas	Kan^R^, *gam-bet-exo*, *cas9*	(Jiang et al. 2015)
pTargetF-adhE2	Derived from pTargetF, *adhE2* knockout vector	This study

*cat*: chloramphenicol acetyl transferase gene; *aadA*: spectinomycin resistance gene; Kan^R^: kanamycin resistant strain; *gam-bet-exo*: Red recombinase genes; *cas9*: Cas9 protein coding gene

### Cell culture and fermentation conditions

For genetic modification, *E. coli* strains were grown aerobically at 37 °C in Luria–Bertani (LB) medium (10 g/L tryptone, 5 g/L yeast extract, 10 g/L NaCl) supplemented with kanamycin (50 μg/mL) when necessary. The strains were preserved in 15% glycerol at −80 °C. The cryopreserved cells were first grown overnight on LB plates, after which fresh colonies were picked and used directly to inoculate LB medium, followed by overnight culture at 37 °C at 200 rpm.

Tube fermentation was performed in a sealed 10 or 50 mL polypropylene conical tube (BD Biosciences, San Jose, CA) containing various volumes of the medium, which was slightly modified from M9Y medium (Dong et al. 2017) (M9 medium + 2 g/L yeast extract + 20 g/L glucose). Cells were cultured at 37 °C with constant shaking at 200 rpm for 48 h or longer where indicated. Samples comprising 0.5 mL of the fermentation broth were harvested every 24 h, and centrifuged at 19,216 × *g* for 1 min. The supernatant was filtered through a 0.22 µm pore-size filter membrane (nylon), and transferred into 2 mL HPLC vials for analysis of residual sugar and metabolites.

Fermentations were conducted in 1 L Infors HT bioreactors containing 0.9 L fermentation medium (M9 medium + 5 g/L yeast extract + 60 g/L glucose) with air sparging. The agitation speed was set to 200 rpm and the pH was maintained at 6.8 by the automatic addition of 5 M NaOH. During fermentation process, 1 mL of the fermentation broth was withdrawn for analysis every 24 h, 0.5 mL of which was used for cell growth monitoring, while the other 0.5 mL was used for HPLC as described above.

The final OD_600_ of the butanol- and butyrate-producing strains after overnight culture in LB medium were not the same but they did not differ much. The two cultures were inoculated in a designed OD ratio. To calculate the precise volume of butanol- and butyrate-producing strains to be inoculated to achieve a designed inoculum ratio, we used the system of equations:

1 $${\text{Ratio of }}\frac{{\text{Butyrate strain}}}{{\text{Butanol strain}}} = \frac{XV1}{{YV2}}$$

2 $$a = XV1 + YV2$$

where *X* represents the OD of the butanol-producing strain, *Y* represents the OD of the butyrate-producing strain, while *V*1 and *V*2 represent the respective volumes of the butanol- and butyrate-producing strains to be inoculated.

The starting optical density for inoculation was set at 0.2 and 0.4 for tube and bioreactor fermentation, respectively. Therefore, the value of $a$ in Eq. (2) is the total OD to be inoculated, which can be 1, 2, 3, 4, 6, or 400 when the fermentation broth is 5, 10, 15, 20, 30 mL in tubes, or 1000 mL in the bioreactor, respectively. For example, when using a ratio of 1:4 in a bioreactor experiment with 1000 mL of fermentation broth, the initial inoculum volume of the two strains was determined using the following system of equations:

3 $$\frac{1}{4} = \frac{XV1}{{YV2}}$$

4 $$400 = XV1 + YV2$$

### Production of BB from glucose

For BB production, overnight cultures of the butyrate-producing strain EB243ΔadhE2::yciAh and butanol-producing strain EB243 (at an inoculation ratio of 1:4), were seeded into 15 mL M9Y medium in a sealed 50 mL conical polypropylene tube. Additionally, 5 g/L of LCS (recombinant lipase from *Candida* sp., expressed in *Aspergillus niger*; Novozymes Lipozyme® CALB, Sigma-Aldrich) was used to convert the butanol and butyrate into BB. CALB should be added when the substrate is available. The concentrations of butyrate and butanol after 8, 10, and 12 h of fermentation were then determined. To extract the produced BB, 15 mL hexadecane (Sigma-Aldrich) was added to each 50 mL conical polypropylene tube. The cultures were incubated in a rotary shaking incubator at 200 rpm and 37 °C for 72 h. Every 24 h, 1 mL of culture was collected for analysis of metabolites and residual sugar. At the same time, 1 mL of the hexadecane layer was also sampled to detect the concentration of the produced BB. The produced BB is efficiently extracted into the organic phase, as a previous study demonstrated that the partitioning coefficient for BB in the hexadecane/aqueous system is more than 300 (Zhang et al. 2017). To ensure the detection of all BB, the concentration of butyl butyrate in the aqueous phase was also measured, but BB could not be detected and its aqueous solubility can thus be neglected. All data on the concentration of BB therefore refer to what was detected in the organic phase. Since the volume of the organic phase and aqueous culture broth was 1:1, the concentration of BB in the organic phase was equal to the concentration produced in the aqueous culture broth.

### Genetic manipulation and strain development

The simultaneous knockout of *adhE2* and the integration of *yciAh* with a strong RBS and miniPtac promoter in the chromosome of strain EB243 was carried out using a published CRISPR/Cas9 (Jiang et al. 2015). Briefly, a pTargetF-derivative plasmid harboring a designed N20 DNA sequence from the genomic target gene and the corresponding homologous fragment was used to co-transforme fresh *E. coli* competent cells along with the pCas plasmid, which expresses Cas9 protein and Red recombinase. The correct transformants were screened by colony PCR and confirmed by DNA sequencing. Inducing pCas with IPTG results in cells free from the pTargetF vector, while pCas can be cured by cultivating mutant cells at an elevated temperature since pCas is temperature-sensitive. For *yciAh* integration, the primers pTargetF-adhE2N20-1/pTargetF-2 were used to amplify pTargetF-adhE2 containing the designed N20 sequence, while the primer pairs adhE2-up-F/adhE2-up-R and adhE2-down-F/adhE2-down-R were used to amplify the homologous arms. Similarly, yciAh-F with adhE2-up-R half homologous sequence and yciAh-R with adhE2-down-F half homologous sequence was used to amplify the *yciAh* gene for chromosomal integration. Then, the three fragments were fused to form the homologous sequence. Subsequently, pTargetF-adhE2, and the homologous sequence were introduced into the EB243 strain harboring pCas. The resulting mutant strain was verified by colony PCR using the primer pair adhE2-up-F/adhE2-down-R. In the end, we obtained a plasmid-free strain by applying the curing strain strategy as described above. The integrated sequences were amplified from the constructed pAC2-based plasmids (Table 1), constructed using the Gibson assembly kit (New England BioLabs, Beijing, China).

### Analytical methods

To assess strain growth, the optical density at 600 nm (OD_600_) was measured using a UV-2802PC; spectrophotometer (Unico, Shanghai, China). The concentrations of butyrate, butanol, and glucose in the fermentation samples were measured by HPLC using an Agilent 1260 system (Agilent Technologies, Santa Clara, CA, USA), equipped with an HPX-87H column (Bio-Rad Laboratories, Inc., Richmond, CA, USA) kept at 55 °C, with 5 mM H_2_SO_4_ at a flow rate of 0.5 mL/min as the mobile phase. The injection volume was 10 μL injection. For measurement of BB production, samples were taken from the solvent phase during fermentation, filtered, and immediately analyzed on a GCMS-QP2010 Ultra (Shimadzu, Japan) system equipped with a DB-5 ms column (30 m length, 0.25 mm inside diameter, 0.25 μm thickness, Agilent, USA). The flow rate of the helium carrier gas was 1 mL /min. The interface and ion source temperatures were set to 250 and 200 °C, respectively. The electron impact voltage was set to 70 eV. The *m/z* range was 35–500. The column temperature was initially set to 100 °C, after which it was increased to 250 °C at a rate of 20 °C/min, where it was held for 5 min.

### Statistical analysis

Statistical analysis using Student’s *t*-test and plotting of diagrams was performed in Origin software. *P* values of < 0.05 were considered to indicate statistical significance.

## Results and discussion

### Construction of a butyrate-producing strain

A butyrate-producing strain was constructed starting from the chromosomally engineered *E. coli* strain EB243, which is capable of efficiently producing butanol from glucose (Dong et al. 2017). Since the production of butyrate and butanol diverges at the node of butyryl-CoA, a straightforward engineering strategy would be to block the butanol synthesis of strain EB243 while simultaneously introducing a suitable enzyme capable of converting butyryl-CoA into butyrate. Thioesterase, butyrate kinase, and phosphate butyryltransferase are all capable of catalyzing this reaction. Therefore, three acyl-CoA thioesterase genes (*yciA* and *tesB* from *E. coli*, as well as *yciAh* from *H. influenzae*), one phosphate butyryltransferase gene (*ptb* from *C. acetobutylicum*), and two butyrate kinases genes (*buk* and *buk2* from *C. acetobutylicum*) were selected for testing.

The starting strain *E. coli* EB243 (Dong et al. 2017) only generated 0.25 g/L butyrate after 72 h of fermentation, suggesting a very weak butyrate production ability. This is due to the string activity of aldehyde/alcohol dehydrogenase (AdhE2), which was introduced for butanol production. Therefore, *adhE2* had to be deleted and genes responsible for butyrate formation had to be introduced. Accordingly, *adhE2* was first deleted to form the strain EB243ΔadhE2. Subsequently, the aforementioned selected genes from various sources were expressed to increase the titer and yield of butyrate. Plasmids harboring the genes of interest were first constructed in *E. coli* DH5α, screened by colony PCR, and verified by sequencing before transformation of the EB243ΔadhE2 strain.

### Production of butyrate in tube and bioreactor fermentation

**Fig. 2:**
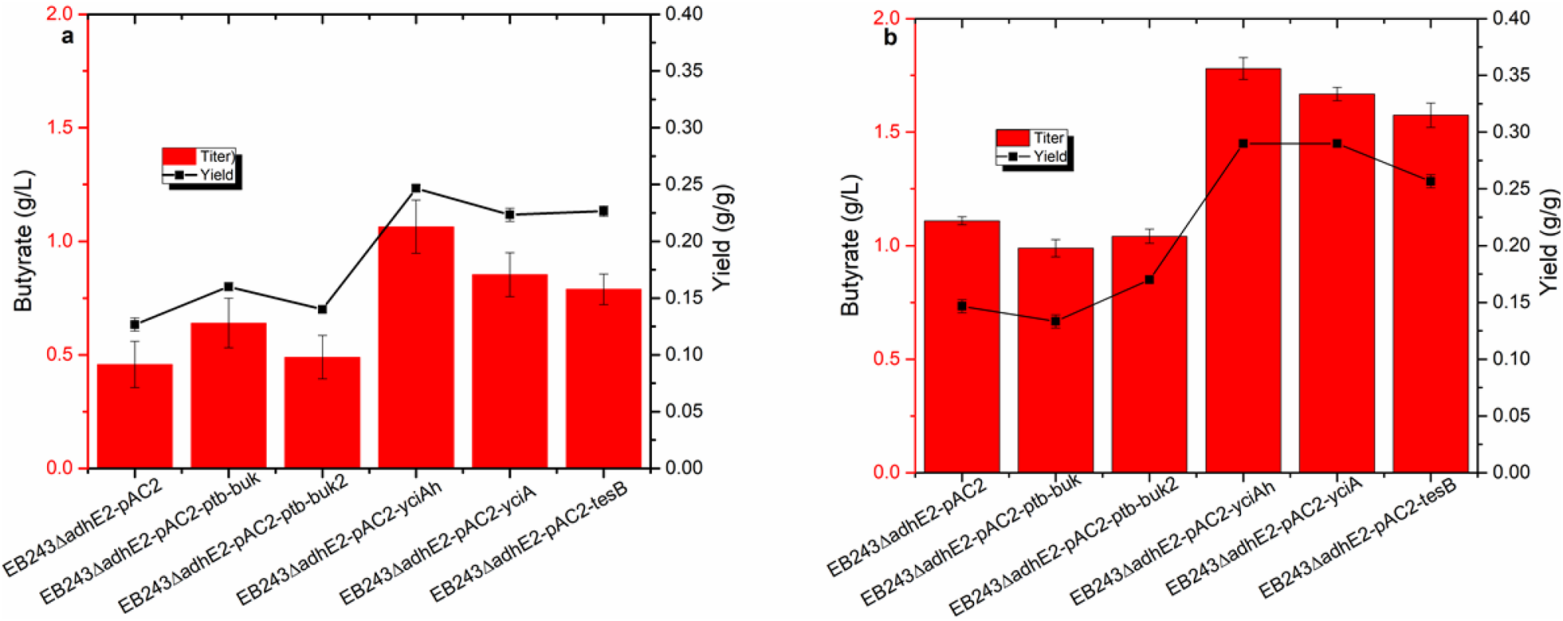
Effects of overexpressing different butyrate-biosynthesis genes on the product titer. In **a** the strains were cultured in 10 mL polypropylene centrifuge tubes containing 5 mL of M9Y medium, while in **b** the strains were cultured in 50 mL polypropylene centrifuge tubes containing 5 mL of M9Y medium. The designation “*yciAh*” represents a synthesized version of the *yciA* gene from *Haemophilus influenzae*. The data represent the means ± SD from three biological replicates

To test if the candidate genes can increase butyrate production, three single genes (*yciA*, *tesB*, and *yciAh*) encoding thioesterase and two gene pairs (*ptb-buk*, and *ptb-buk2*), respectively encoding phosphate butyryltransferase and butyrate kinase, were cloned into the pAC2 plasmid and expressed in strain EB243ΔadhE2. The resulted strains were individually cultured in 5 mL M9Y medium in 10 mL polypropylene centrifuge tubes with appropriate antibiotics. The strain containing the synthesized *yciAh* gene from *H. influenzae* manifested the highest butyrate production of 1.06 g/L in 72 h, with a yield of 0.29 g/g glucose, while the butyrate production of the strains containing all the other genes was below 1 g/L (Fig. 2a).

The supply/consumption of NADH is balanced in strain EB243, which is capable of efficiently producing butanol. However, once strain EB243 was engineered from butanol production to butyrate production, the metabolic changes resulted in an excess of NADH. This is because the NADH consumed by aldehyde/alcohol dehydrogenase (encoded by *adhE2*) in strain EB243, cannot be recycled in the *adhE2*-deleted strain EB243ΔadhE2, and the pathway for butyrate production does not require NADH. To recycle the NADH and thus continue the fermentation, oxygen needs to be supplied for butyrate production. In fact, strain EB243ΔadhE2 did not grow, nor produce butyrate, under anaerobic conditions (data not shown).

Subsequently, the production of butyrate by the six constructed strains in 50 mL polypropylene conical tubes containing 5 mL M9Y medium was quantified and compared. All strains produced a higher titer of butyrate (Fig. 2b) compared to the titer observed when the fermentation was performed in 10 mL polypropylene centrifuge tubes (Fig. 2a). Strain EB243ΔadhE2*-*pAC2-yciAh produced the highest butyrate titer among all recombinant strains. After changing the culture volume, additional tube fermentation experiments were performed to study the effect of aeration on butyrate production. It was found that strain EB243ΔadhE2*-*pAC2-yciAh produced a maximal butyrate titer of 3.5 g/L with a yield of 0.34 g/g glucose when 30 mL of M9Y medium was used in a 50 mL tube (Additional file 1: Fig. S1c). This suggests that moderate aeration is needed for butyrate production.

### Batch fermentation for butyrate production

The *yciAh* gene, which resulted in the best butyrate production in tube-scale fermentations, was integrated chromosomally to obtain an antibiotic-independent fermentation strain. The resulting strain EB243ΔadhE2::yciAh was further subjected to bioreactor fermentation to evaluate its butyrate production ability. As the butyrate production is strongly related to oxygen availability, the fermentation system was aerated at rates of 0.3, 0.5, 0.75, and 1 vvm (volume of gas per volume of liquid per minute) (Additional file 1: Fig. S2), which resulted in final OD_600_ values of 16.2, 17.2, 13.2, and 13.9, respectively (Additional file 1: Fig. S2a). Strain EB243ΔadhE2::yciAh produced 12.4 g/L of butyrate under the aeration rate of 0.5 vvm. A promising butyrate yield of 0.46 g/g of glucose (93.9% of the theoretical yield) and a productivity of 0.17 g/L/h were achieved after 72 h, which was the highest yield reported for *E. coli* to date (Wang et al. 2019). A higher air flow of 0.75 or 1 vvm did not favor butyrate production.

Normally, butyrate is produced by clostridia under anaerobic conditions through the *ptb*-*buk* pathway, while thioesterase genes are commonly found in aerobic microorganisms. Since aerobic conditions are required for the production of butyrate by engineered *E. coli,* the thioesterase may function better than *ptb*-*buk* under aerobic conditions. This is likely the reason why a single thioesterase out-performs *ptb*-*buk* for butyrate production. Although a certain amount of butyryl-CoA may be produced by fatty acid degradation (FAD) (Iram and Cronan 2006), it is questionable whether this contributed significantly to the produced butyrate. However, since it is inordinately challenging to calculate how much fatty acids are degraded to yield butyryl-CoA in a growing *E. coli*, and the fact that no external fatty acids were added to the medium, the quantity of butyrate that may have been derived from FAD was not evaluated in this study.

### Co-production of butanol and butyrate by the consortium

**Fig. 3:**
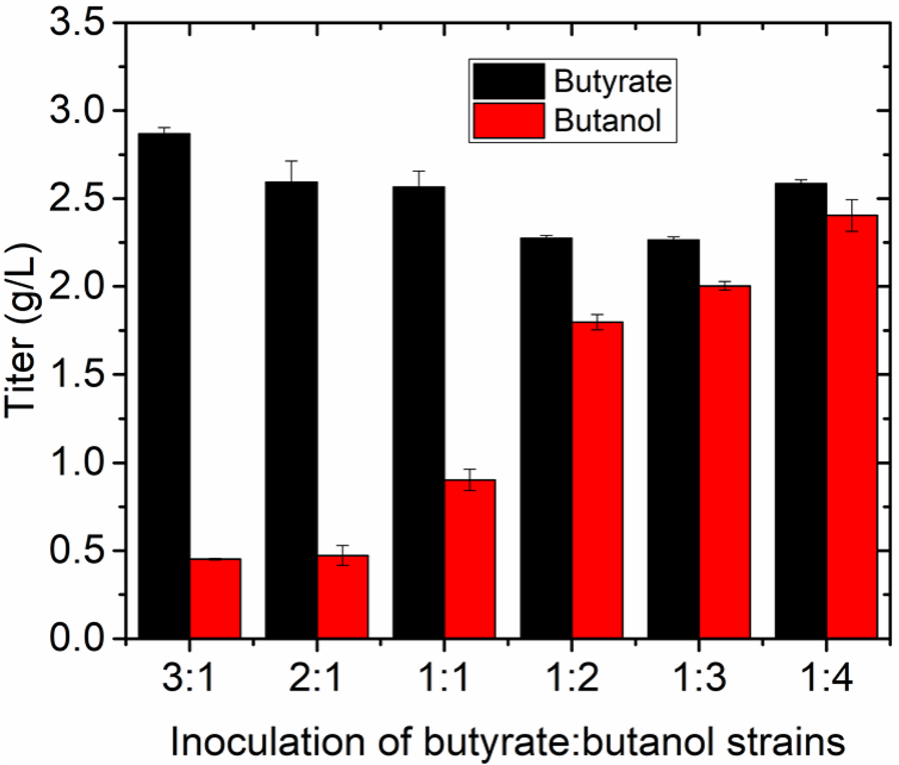
Production of butyrate and butanol using consortia with varying inoculation ratios of the butyrate- and butanol-producing strains. The titers of butyrate and butanol were measured after 72 h of tube fermentation. The data represent the means ± SD from three biological replicates

Using the butyrate-producing strain EB243ΔadhE2::yciAh and the butanol-producing strain EB243 (Dong et al. 2017), a microbial consortium was built to simultaneously produce the butanol and butyrate required for BB biosynthesis. However, the constructed consortium could not produce butyrate under anaerobic conditions, while under aerobic conditions, it could produce butyrate but the butanol production would be impaired. Considering the demand of moderate aeration for butyrate biosynthesis as described above, and the anaerobic conditions suitable for butanol fermentation (Dong et al. 2017), the mismatched oxygen demand would be a challenge for the synchronous production of butyrate and butanol. To address this challenge, the ratio of the butyrate- and butanol-producing strains was altered to enable the consortium to produce both butyrate and butanol under moderately aerobic conditions. The ratio of 1:4 (butyrate strain: butanol strain) was shown to be the best for the simultaneous production of butyrate and butanol in tube fermentation. Using this strategy, the titer of butyrate and butanol reached 2.5 and 2.4 g/L, respectively (Fig. 3 and Additional file 1: Fig. S3f). Notably, butanol production under aerobic conditions increased along with the increased ratio of the butanol-producing strain in the consortium, suggesting that altering the ratio of the consortium is an effective approach to optimize production.

### In situ production of BB using the microbial consortium in the presence of lipase

#### Batch fermentation for the direct in situ production of BB

The production of BB by engineered *E. coli* is generally low (at mg/L levels) even with the supplementation of exogenous substrates. In one study, alcohol acyltransferase (AAT) from *Fragaria ananassa*, a cultivated strawberry, was successfully expressed in *E. coli*, and the resulting strain subsequently cultured with exogenous BB precursors. However, only 0.28 mg/L BB was produced when 1 g/L butanol and 3 g/L butyryl-CoA were added to the system (Horton and Bennett, 2006). In 2014, various modules related to the production of alcohols, along with an alcohol O-acyltransferase (ATF1) from *S. cerevisiae*, which is known to catalyze the last step of ester biosynthesis, were designed and introduced into *E. coli*. However, in spite of many esters produced in the mixture, no BB was detectable. Based on the idea that this strategy could provide butyryl-CoA, the process was supplemented with 3 g/L of butanol, which resulted in a low BB titer of 14.9 mg/L (Rodriguez et al. 2014). Another study aimed to engineer *E. coli* to produce BB via fermentative biosynthesis (Layton and Trinh 2014). In said study, the enzymatic ester pathway with the AAT sub-module from *Fragaria* *ananassa* as introduced into *E. coli* to generate alcohol and acyl-CoAs molecules. However, no BB was detected among the produced esters, which may have been caused by insufficient butanol in the system. Although BB could finally be produced after adding 2 g/L butanol, the titer of 36.8 mg/L was low. A further study demonstrated the prospect of constructing biotechnological carboxylate-to-ester platforms. To implement this, a modular *E. coli* chassis cell was precisely assembled using heterologous pathways comprising an acid to acyl-CoA synthesis sub-module (acyl-CoA transferase), an acyl CoA and alcohol condensation sub-module (alcohol acyltransferase), and an alcohol production sub-module. When the strain was fermented with glucose to form a combinatorial biosynthesis of fermentative esters, 2 g/L butyrate was supplemented to the fermentation medium to reinforce the CoA molecule. However, only 47.6 mg/L BB was produced (Layton and Trinh 2016).

The reason for the low titers produced by these engineered *E. coli* strains is difficult to fully elucidate due to insufficient information on the characteristics of alcohol acyltransferase. Moreover, the biotechnological production of BB is also close linked with the supply of intrinsic precursors such as butyryl-CoA and butanol. Preferably, both substrates should be produced at a ratio of 1: 1 for efficient conversion of sugars into BB. However, this is a problem in *E. coli*, since butyryl-CoA and butanol are produced in a complex and interlinked metabolic pathway, making it challenging to balance their ratio.

**Fig. 4:**
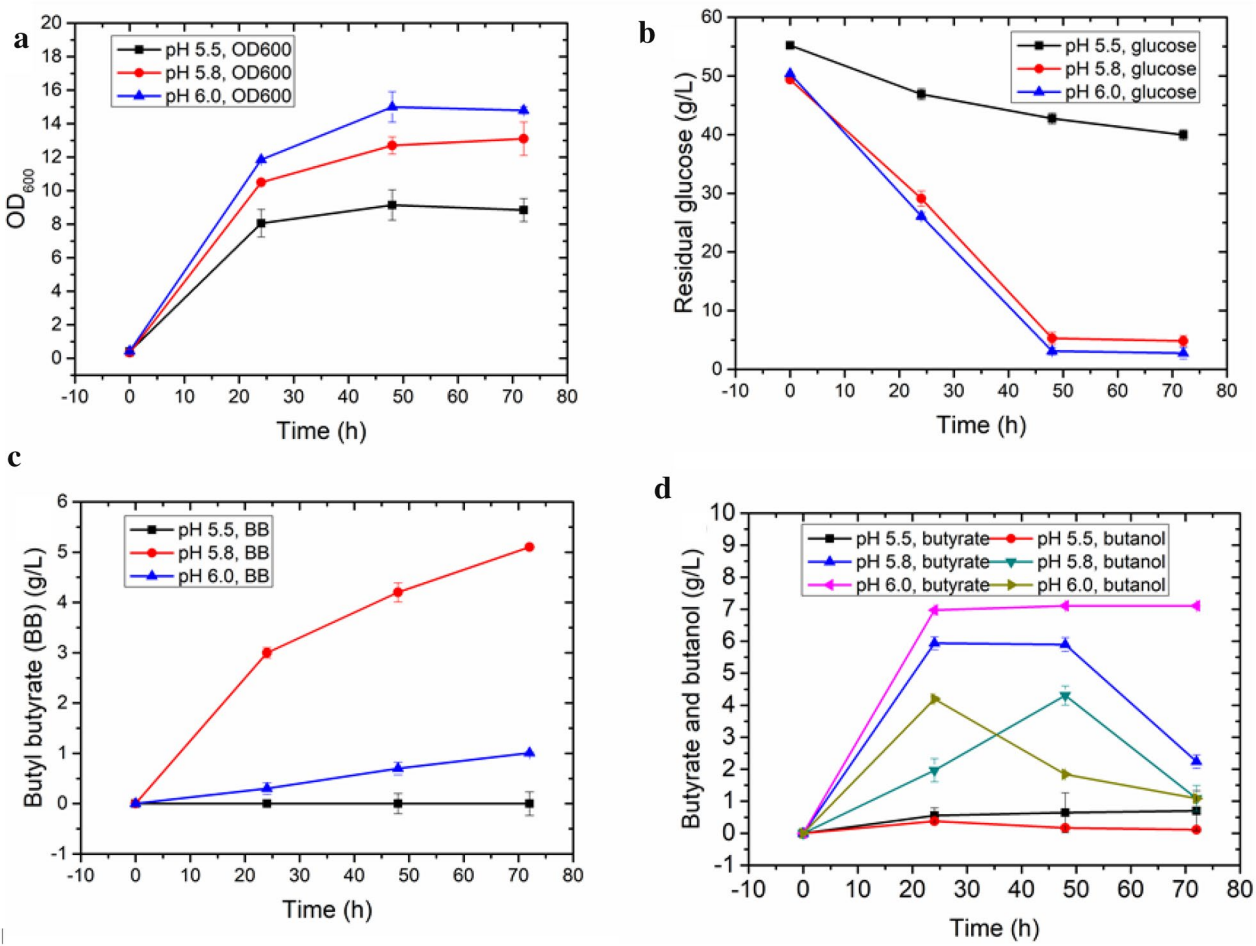
Bioreactor batch fermentation profiles of the two strains for butyl butyrate production at different pH values. The strains were seeded at an inoculation ratio of 1:4 (butyrate-producing strain (EB243Δ*adhE2::*yciAh): butanol-producing strain (EB243) in modified M9Y medium supplemented with 50 g/L glucose. The system was composed of the fermentation broth and hexadecane as extractant at a volume ratio of 1:1. **a** Optical density at 600 nm (OD_600_), **b** glucose consumption, **c** butyl butyrate produced during the fermentation, and **d** butyrate and butanol. All parameters were assessed at pH 5.5, 5.8, and 6.0. Butyrate, OD_600_, butanol, butyl butyrate, and glucose were measured every 24 h. Error bars indicate the standard deviations from three biological replicates

To address this challenge, batch fermentation of a consortium comprising separate engineered butyrate- and butanol-producing strains was carried out in bioreactors with 0.5 L of modified M9 medium and 0.5 L hexadecane as an extractant, allowing the in situ removal of BB from the aqueous phase to avoid potential product inhibition. The pH was maintained at 6, and air was sparged at a rate of 0.5 vvm for aerobic growth. Additionally, an agitation speed of 200 rpm was maintained, since thoroughly mixing the butyrate and butanol is essential for the lipase-catalyzed esterification reaction (Additional file 1: Fig. S4). Under these conditions, 1.1 g/L BB was produced at the end of the fermentation (Fig. 4c). While 7.1 g/L butyrate was still present in the fermentation broth at this point, the residual butanol concentration was only 1 g/L (Fig. 4d). Hence, insufficient butanol can explain the low BB titer. Additionally, as pH may affect the dissociation status of butyrate, the effect of pH was also further investigated.

## Optimization of pH control for improved BB production

Butyrate is present in un-dissociated at low pH, which may favor the esterification of butyrate with butanol to produce BB (Harroff et al. 2019; Zhang et al. 2017). However, low pH may severely impair bacterial cell growth and lead to poor fermentation performance (Maddox et al. 2000). Therefore, the effect of pH on the production of BB by the consortium in the bioreactor was investigated.

At pH 4.5 and 5, the consortium could not grow (data not shown), while at pH 5.5, the microbial consortium grew moderately, but there neither butyrate nor butanol was detectable (Fig. 4d). This is closely related to the poor growth of the strains, leading to slow glucose utilization, which was reflected in the high residual concentration (Fig. 4b). This means that pH lower than 5.5 is not suitable for the simultaneous biosynthesis of butyrate and butanol in *E. coli* (Fig. 4). To tackle this challenge, a parallel experiment was performed at pH 5.8. At this pH, the consortium was able to grow well (Fig. 4a), and almost all the glucose was consumed (Fig. 4b). Additionally, 2.2 g/L butyrate along with 1.0 g/L butanol remained in the fermentation medium at the end of fermentation. Overall, the consortium produced 5.1 g/L BB (Fig. 4c).

## Increasing the ratio of the butanol-producing strain favored BB production

**Fig. 5:**
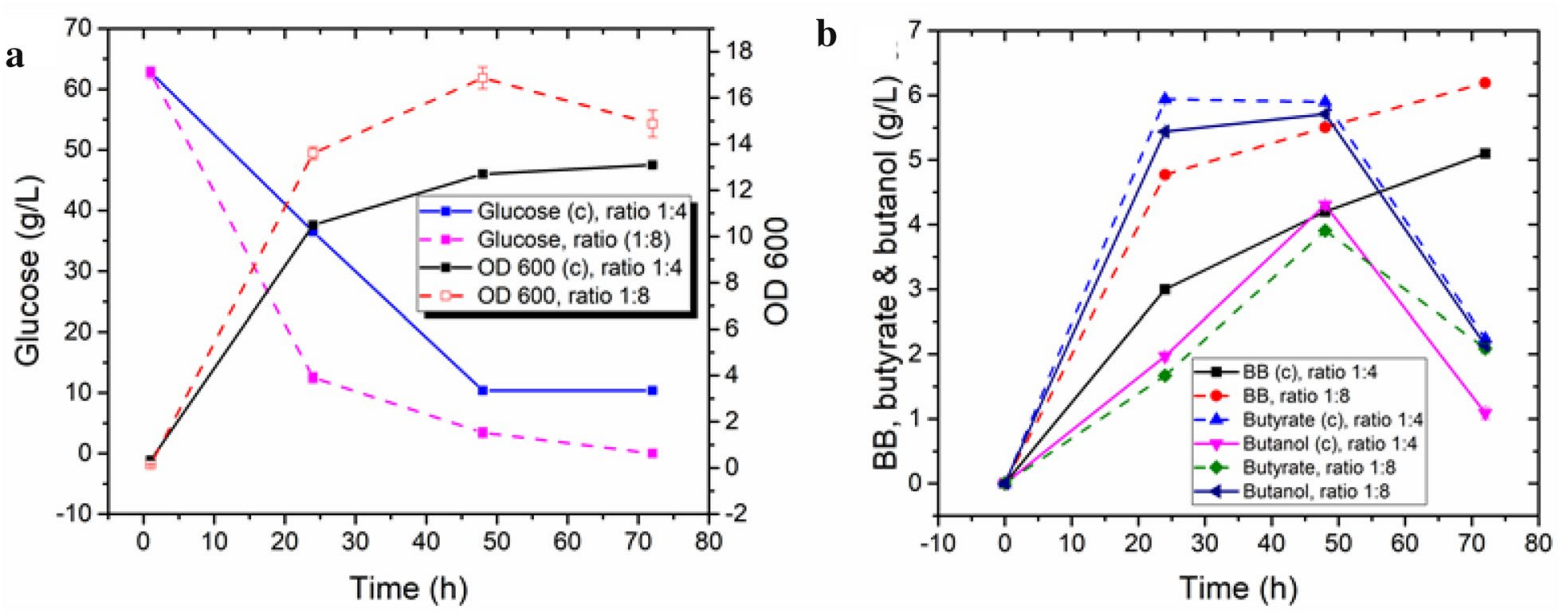
Fermentation profiles of consortia with inoculation ratios (butyrate-producing strain: butanol-producing strain) of 1:4 (control, solid line) and 1:8 (dash line). **a** Profiles of optical density (OD_600_) and glucose consumption. **b** Time-profiles of the butyrate, butanol, and butyl butyrate titers. The data represent the means ± SD from three independent measurements

In the optimization experiments, the concentrations of butanol in the fermentation broth were always lower than the corresponding concentrations of butyrate. Therefore, BB synthesis was likely limited by the supply of butanol. Since the ratio of butanol to butyrate is the key factor determining the final production of BB, it can be controlled by adjusting two process variables. One is the initial inoculation ratio of the butanol- and butyrate-producing strains, and the other is aeration, which affects both butanol and butyrate production. In tube fermentation, a ratio of 1:4 of the butyrate- and butanol-producing strains was shown to generate a comparable amount of butyrate and butanol. However, applying such a ratio in the fermenter left a final titer of 7.1 g/L of unconsumed butyrate in the fermentation due to insufficient butanol. Since the conditions in the fermenter do not favor butanol production, the initial inoculum of the butanol-producing strain needed to be increased. As a consequence, increasing the ratio of the butyrate- and butanol-producing strains from 1:4 to 1:8 increased the BB production to 6.1 g/L. However, this was accompanied by a low yield of 0.09 g/g glucose, whereby the residual concentrations of butyrate and butanol were roughly the same (Fig. 5b). Compared with the control consortium inoculated at a ratio of 1:4 (butyrate strain: butanol strain), it is evident that the consortium inoculated at a ratio of 1:8 grew significantly better (Fig. 5a). Once the inoculum ratio was optimized and fixed at the fermentor scale, the next key factor to be optimized was aeration. Since aeration favors butyrate production but inhibits butanol production, finding a balanced aeration strategy was challenging.

## Optimizing aeration to improve BB synthesis

Since butyrate- and butanol-producing *E. coli* strains were required to construct a “diamond-shaped” consortium for efficient BB production, optimal aeration is necessary to produce a balanced ratio of the two precursors. When the consortium inoculated at a ratio of ratio 1:4 was grown under fully aerobic conditions, at 0.5 vvm, the butyrate titer reached 5.9 g/L in 24 h of fermentation, while the butanol titer only reached 1.9 g/L under the same conditions. By increasing the ratio from 1:4 to 1:8, the butanol titer reached 5.4 g/L, but there was only 1.6 g/L of butyrate in the first 24 h of the fermentation. This indicates that both strains in the consortium still need optimal aeration conditions to efficiently balance the butanol and butyrate supply, thereby increasing BB production. Since our previous research on butanol production indicated the need for microaerophilic conditions in the beginning of fermentation to facilitate cell growth, a two-stage aeration strategy for BB production was proposed. In this two-stage strategy, relatively high aeration is provided during the first stage to promote the growth of both butyrate- and butanol-producing strains, while also facilitating butyrate production. Subsequently, the aeration might be decreased at a certain time point to favor butanol production, while still sustaining butyrate production. Such a two-stage aeration strategy is expected to result in a better balance between the different oxygen demands of the butyrate- and butanol-producing strains, thus achieving higher BB production.

**Fig. 6:**
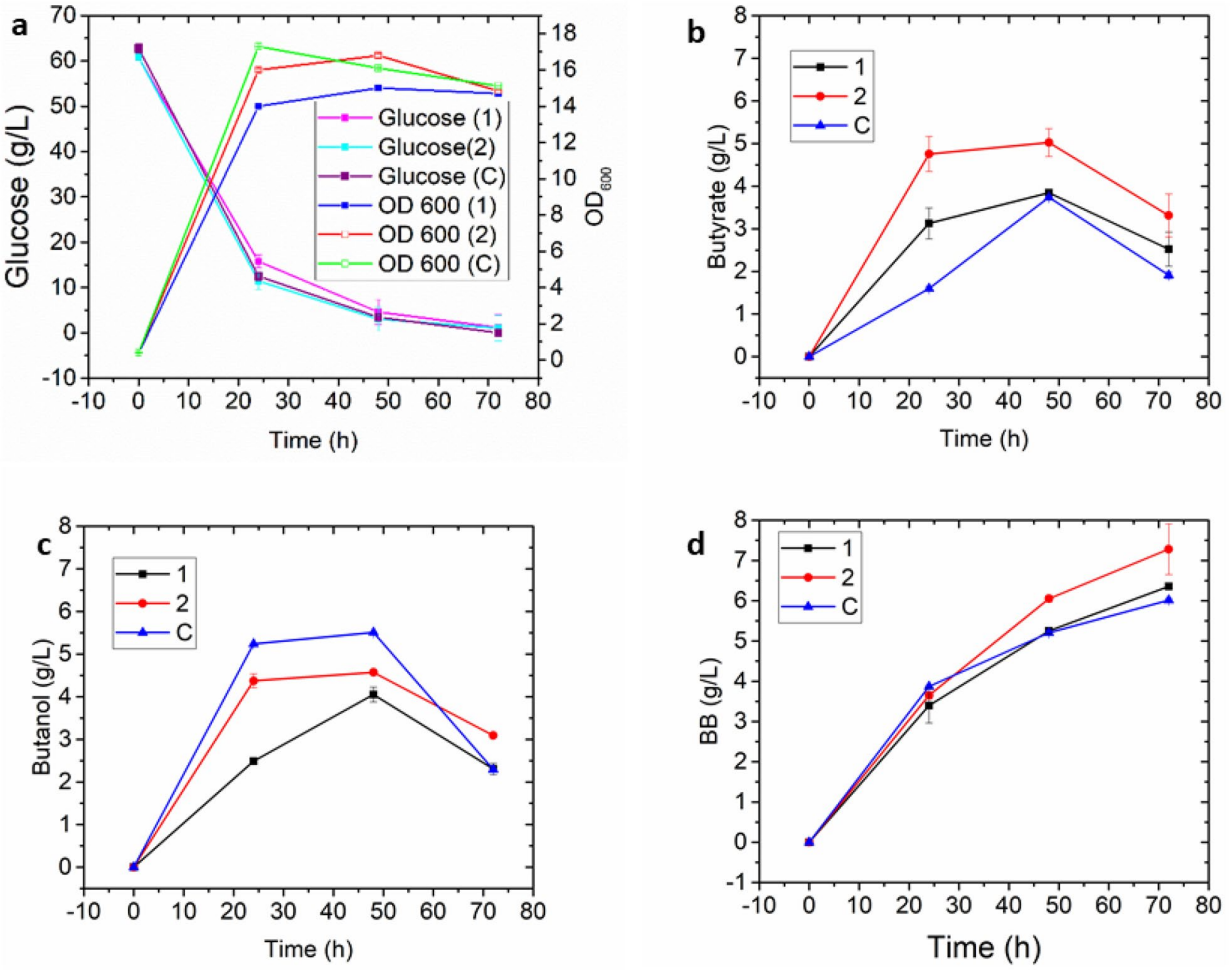
Batch fermentation profiles of the microbial consortium for butyl butyrate production with different aeration speed control strategies. The consortium strains were inoculated at a ratio of 1:8 (butyrate strain: butanol strain) and cultured at 200 rpm, pH 5.8. **C:** control, aerated with 0.5 vvm during the whole fermentation process. **1:** aerated with 0.5 vvm in the first 24 h, then shifted to 0.1 vvm. **2:** aerated with 0.5 vvm in the first 36 h, then shifted to 0.1 vvm. The data represent the means ± SD from three biological replicates

When aerobic conditions were applied in the first 24 h (strategy 1), the excess NADH could be oxidized, resulting in efficient butyrate production. Since the consortium requires butanol and the corresponding strain does not tolerate excess aeration, an anaerobic stage would favor the production of butanol. Nevertheless, the BB titer produced via this strategy was not significantly increased compared to an entirely aerobic process (6.35 vs. 6.1 g/L). Similar to the glucose consumption of the consortium grown in the one-stage fermentation (Fig. 5a), the consortium grown at an air flow rate of 0.5 vvm in the first 24 h (strategy 1) or 36 h (strategy 2), then shifted to 0.1 vvm in the second stage of the fermentation up to 72 h, consumed almost all glucose by the end of the fermentation (Fig. 6a). As shown in Fig. 6d, the consortium grown using strategy 2 produced 7.2 g/L BB with a yield of 0.12 g/g of glucose, compared to 6.35 g/L BB with a yield of 0.11 g/g of glucose in strategy 1. Interestingly, the consortium grown using strategy 2 was still able to maintain the momentum to produce butanol (Fig. 6c) and butyrate (Fig. 6b), compared with the consortium without two-stage aeration control. These results demonstrated that the approach not only increased the BB titer but also slightly increased the yield. However, the yield obtained is much lower than the theoretical yield of BB from glucose (0.4 g/g). This yield limitation is most likely related to aeration, and needs further investigation beyond the scope of this study. As the ratio of initial inoculum and aeration both affect the production of butyrate and butanol, iterative optimization of these two factors is required to efficiently improve BB biosynthesis at a different scale of fermentation, as shown in this study.

Very recently, Cui et al. developed a microbial consortium to produce BB. The consortium comprises two different species, *C. beijerinckii* BGS1 and *C. tyrobutyricum* ATCC25755, which could produce 6.8 g/L butanol and 9.7 g/L butyrate, respectively, during the co-culture fermentation process. After adding lipase, 5.1 g/L of BB with a yield of 0.068 g/g was produced. It is likely that the amount of butanol produced by *C. beijerinckii* BGS1 was insufficient for the consortium to achieve a high titer. Surprisingly, the BB yield of this *Clostridium* co-culture was 50% lower than the yield achieved in our study (0.12 g/g), although the *Clostridium* co-culture process is performed in an anaerobic process that does not require aeration and vigorous mixing. The low yield might be due to the accumulation of byproducts including acetone and isopropanol, whereas no significant byproducts were detected in our study. Another reason might be the imbalance between butanol and butyrate production in the *Clostridium* co-culture process, which hampers the further improvement of the titer and yield of BB. Moreover, since the *Clostridium* co-culture comprises two strains belonging to two different species, it is difficult, if not impossible, to synchronize the metabolism of these two *Clostridium* strains. By contrast, the two cognate *E. coli* strains in this study are almost identical except for a few genes. This makes it much easier to achieve homogeneity despite different inoculation ratios. Furthermore, *E. coli* fermentation does not require anaerobic handling and can therefore be more cost-effective.

The cognate consortium strategy used in this study corroborates very recent studies that demonstrated the ability of microbial consortia to relieve the metabolic burden of complex pathways when a single strain is used in biotransformations. By segregating biocatalytic pathway into three basic *E. coli* strains, hence constructing a cognate consortium, aliphatic α,ω‐dicarboxylic acids (DCAs) (Wang et al. 2020) and 1,6-hexanediol (HDO) (Zhang et al. 2020) were produced at significantly higher rates than using a monoculture system.

## Conclusions

In this study, we developed a cognate *E. coli* consortium for direct production of butyl butyrate from glucose in a one-pot process. The cognate *E. coli* consortium comprises a butyrate-producing strain and a butanol-producing strain, which share the same pathway upstream of butyryl-CoA. The nearly identical genotype of these two strains lightens the need for manipulation of nutritional conditions for this consortium. The cognate consortium was able to produce 7.2 g/L butyl butyrate from glucose under suitable conditions without the exogenous addition of butanol or butyrate. This is the highest titer of butyl butyrate directly produced from glucose by *E. coli* reported to date, indicating the potential of using engineered *E. coli* consortia for the biotechnological production of esters. Although lipases are arguably the most widely used enzymes for the esterification of carboxylic acids with alcohols, their cost remains a problem, since an optimal balance between the output (titer, yield) and inputs (precursors, enzymes) is needed. To tackle this challenge, future studies should concentrate on overproducing recombinant lipases for selective ester biosynthesis. Moreover, the challenge of different oxygen requirements for butyrate and butanol biosynthesis needs to be addressed to achieve a higher yield of butyl butyrate.

### Supplementary Information

**Additional file 1. **Additional figures and table.

## Data Availability

The supporting documentation showing the findings of this study is available from the corresponding author upon reasonable request.

## Abbreviations

BB: Butyl butyrate
CoA: Coenzyme A
LCS: Recombinant lipase from *Candida* sp., expressed in *Aspergillus niger*; Novozymes Lipozyme® CALB
CRISPR: Clustered Regularly Interspaced Short Palindromic Repeats
GC–MS: Gas chromatography–mass spectrometry
HPLC: High Performance Liquid Chromatography
NADH: Nicotinamide Adenine Dinucleotide
SD: Standard Deviation
OD: Optical Density
pH: Potential of Hydrogen
AAT: Alcohol Acyltransferase
