# Supplementary material for: One-pot production of butyl butyrate from glucose using a cognate “diamond-shaped” E. coli consortium
Source: Bioresour Bioprocess. 2021 Feb 21;8(1):18. doi: 10.1186/s40643-021-00372-8 (PMC10992435; doi:10.1186/s40643-021-00372-8)
Supplement: Supplementary file 1 — Additional file 1. Additional figures and table. [file 40643_2021_372_MOESM1_ESM.docx]

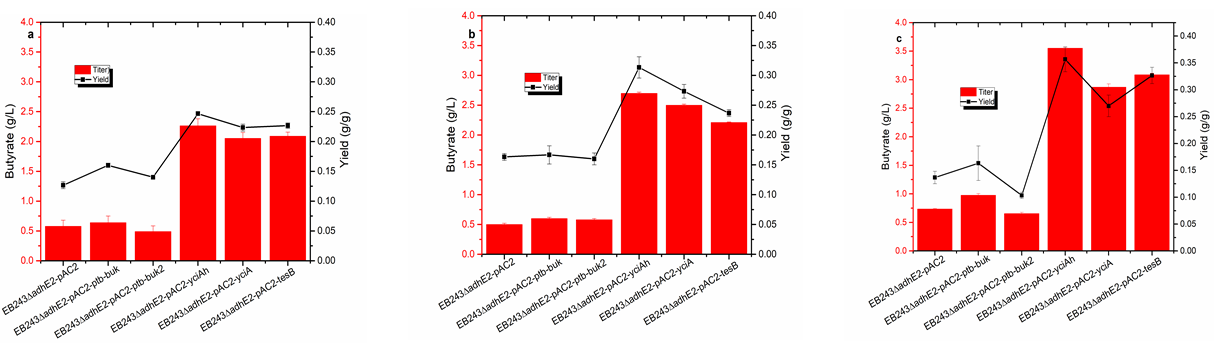


**Fig. S1**Tube-scale fermentation optimization of strains EB243ΔadhE2-pAC2, EB243 ΔadhE2-pAC2-ptb-buk, EB243ΔadhE2-pAC2-ptb-buk2, EB243 ΔadhE2-pAC2-yciAh, EB243ΔadhE2-pAC2-yciA, and EB243ΔadhE2-pAC2-tesB under aerobic conditions. The culture volume in 50-ml polypropylene conical tubes was optimized for adequate aeration and homogenization. The final volume of the cell culture was set at 10 ml in **a,** 20 ml in **b**, and 30 ml in **c**. The black lines represent the yield, and red bars represent the product titer obtained for each strain. The data represent the means ± SD from three biological replicates.

**
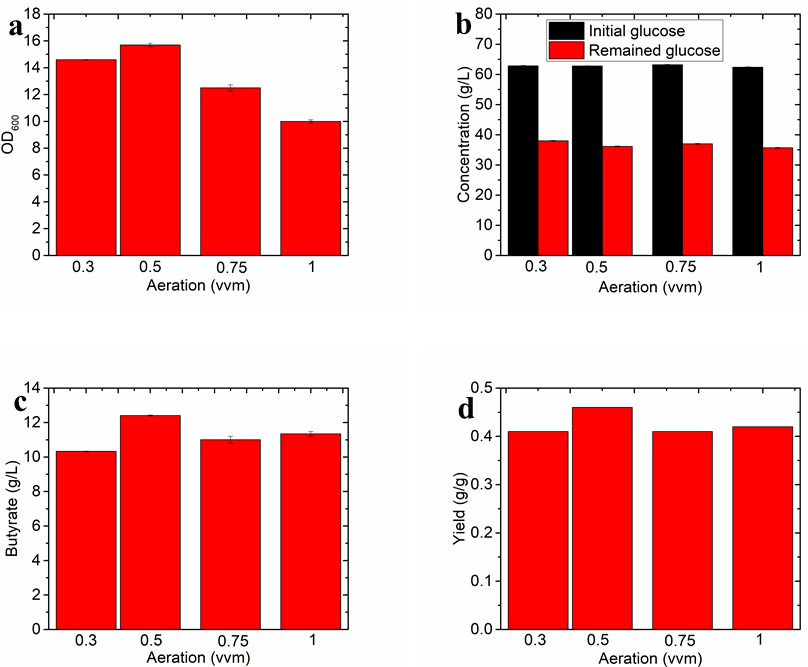
**

**Fig. S2 Growth and fermentation profiles of**E**.**coli**strain** EB243ΔadhE2::yciAh **for butyrate production at** airflow rates of 0.3, 0.5, 0.75, and 1 vvm, respectively. The engineered E. coli strain EB243ΔadhE2::yciAh was grown in modified M9Y medium containing 65 g/L glucose. The pH was controlled at 6.8 during the fermentation. The data represent the means ± SD from two biological replicates at 72 hours of the fermentation.


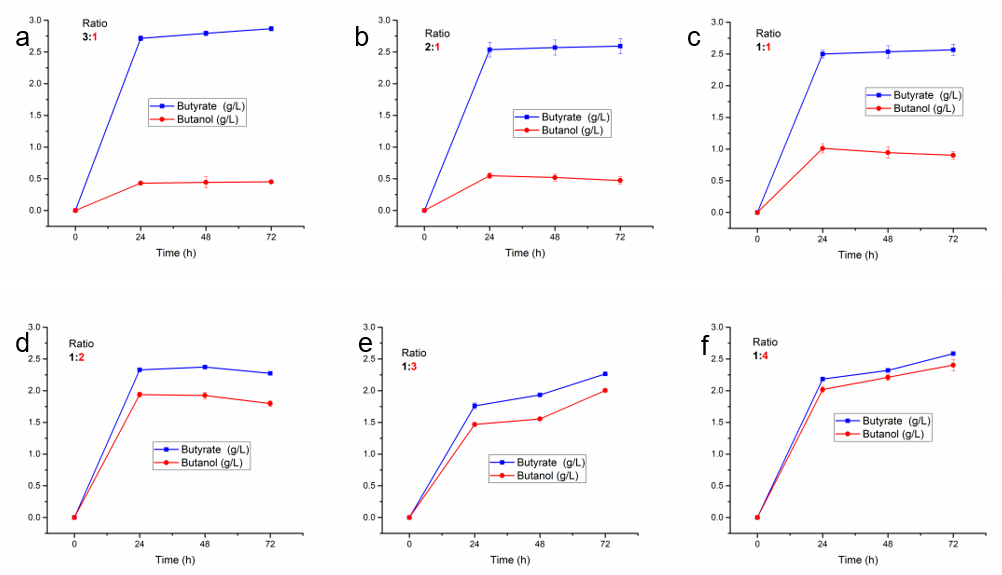


**Fig. S3** **Optimization of butyrate against butanol available in a synthetic microbial consortium comprising two E. coli strains**. Red and black colors denote the butanol- and butyrate-producing strains, respectively, inoculated at ratios of **a** 3:1, **b** 2:1, **c** 1:1, **d** 1:2, **e** 1:3, and **f** 1:4 in tube fermentation of the butyrate producing-butyrate strain (EB243ΔadhE2-yciAh): (butanol-producing strain (EB243). The data represent the means ± SD from three biological replicates.


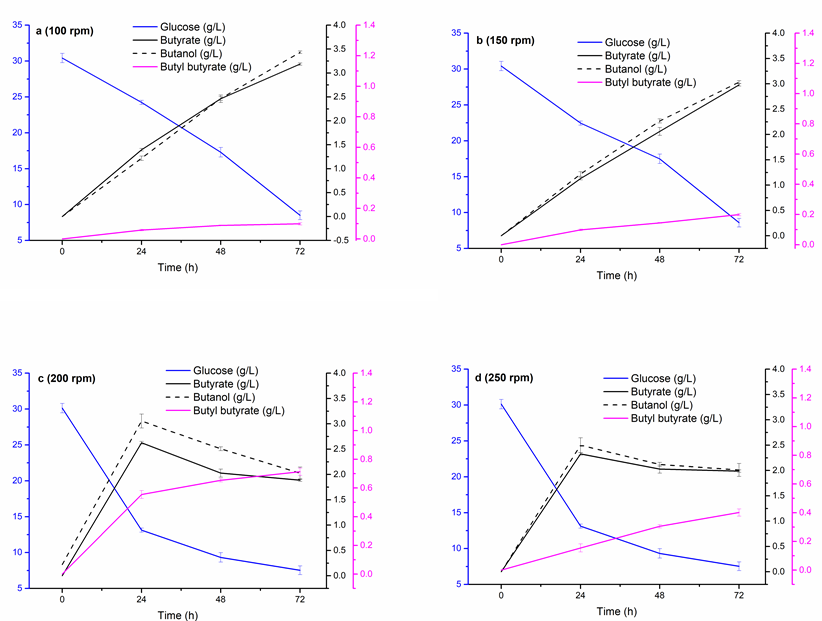


**Fig. S4 Effects of rotation speed on butyl butyrate production in tube-scale fermentations.** Tube- scale fermentations were conducted at (**a**) 100 rpm, (**b**) 150 rpm, (**c**) 200 rpm, and (**d**) 250 rpm. The strains were cultured in modified M9Y medium containing 30 g/L glucose in a semi-sealed 50 mL conical polypropylene tube. The other fermentation condition, including 5 g/L lipase addition, temperature of 37°C, inoculation ratio of two strains: 1:4 (butyrate strain: butanol strain), and 1:1 ratio of fermentation broth: hexadecane as extractant, were kept contstant. The data represent the means ± SD from three biological replicates.

**Table S1 Primers used in this study**

| N^o^ | Primer sequence 5’-3’ | Name |
| --- | --- | --- |
| 1 | ATGTCTACAACACATAACGTCCC | yciA-F |
| 2 | TTACTCAACAGGTAAGGCGCGAGG | yciA-R |
| 3 | ATGTCTGCCAATTTTACTGATAA | yciAh-F |
| 4 | TTACAAGGGTTGTTCTGAAATTAAG | yciAh-R |
| 5 | ATGAGTCAGGCGCTAAAAAATTTAC | tesB-F |
| 6 | TTAATTGTGATTACGCATCACCCC | tesB-R |
| 7 | GTGATTAAGAGTTTTAATGAAATTATC | ptb-F |
| 8 | TTATTTATTGCCTGCAACTAAAGCTGC | ptb-R |
| 9 | ATGTATAGATTACTAATAATCAATC | buk-F |
| 10 | TTATTTGTATTCCTTAGCTTTTTC | buk-R |
| 11 | TGTTGACAATTAATCATCGGCTCGTATAA | buk2-F |
| 12 | TTATTTATATTTTTTAGCTTCTTCTTGTCC | buk2-R |
| 13 | TCCTAGGTATAATACTAGTTGGATCCGCGATTTCTGGGAGTTTTAGAGCTAGAAATAGC | pTargetF-*lld*D2N20-F |
| 14 | CAAATTGGCGTCTCTGATCTGTTG | lldD-arm1-F |
| 15 | CACTTCGGGCTCATGAGCGCTTGTTTCGGTCATGCGTTTTTCTCCCTCGAATGCTCATT | lldD-arm1-R |
| 16 | AATGAGCATTCGAGGGAGAAAAACGCATGACCGAAACAAGCGCTCATGAGCCCGAAGTG | adhE2-F |
| 17 | AGCAACACTTTTAATATTTAATAAATGTTTAGGCTCTACATTTTGCGATACAGAGTTTC | adhE2-Fg1-R |
| 18 | GAAACTCTGTATCGCAAAATGTAGAGCCTAAACATTTATTAAATATTAAAAGTGTTGCT | adhE2-Fg2-F |
| 19 | AGAGGGTTAGGGTGAGGGGGCGCAAACGATCCTTTCGGGCTTTGTTAGCAGCCGGATCT | adhE2-R |
| 20 | AGATCCGGCTGCTAACAAAGCCCGAAAGGATCGTTTGCGCCCCCTCACCCTAACCCTCT | lldD-arm2-F |
| 21 | TGGCGGTACGGGTAAATCTGGTG | lldD-arm2-R |
